# Comprehensive analysis of the transcriptional profile of the Mediator complex across human cancer types

**DOI:** 10.18632/oncotarget.8469

**Published:** 2016-03-30

**Authors:** Isabella Syring, Niklas Klümper, Anne Offermann, Martin Braun, Mario Deng, Diana Boehm, Angela Queisser, Anne von Mässenhausen, Johannes Brägelmann, Wenzel Vogel, Doris Schmidt, Michael Majores, Anne Schindler, Glen Kristiansen, Stefan C. Müller, Jörg Ellinger, David Adler, Sven Perner

**Affiliations:** ^1^ Section for Prostate Cancer Research, University Hospital of Bonn, Bonn, Germany; ^2^ Institute of Pathology, University Hospital of Bonn, Bonn, Germany; ^3^ Center for Integrated Oncology Cologne/Bonn, University Hospital of Bonn, Bonn, Germany; ^4^ Clinic for Urology and Pediatric Urology, University Hospital of Bonn, Bonn, Germany; ^5^ Pathology of the University Hospital of Lübeck and Leibniz Research Center Borstel, Lübeck and Borstel, Germany; ^6^ Department of Oncology, Hematology and Rheumatology, University Hospital Bonn, Bonn, Germany

**Keywords:** transcriptional profile, mediator complex, cancer, oncomine, MED8, Pathology Section

## Abstract

The Mediator complex is a key regulator of gene transcription and several studies demonstrated altered expressions of particular subunits in diverse human diseases, especially cancer. However a systematic study deciphering the transcriptional expression of the Mediator across different cancer entities is still lacking.

We therefore performed a comprehensive *in silico* cancer *vs*. benign analysis of the Mediator complex subunits (MEDs) for 20 tumor entities using Oncomine datasets. The transcriptional expression profiles across almost all cancer entities showed differentially expressed MEDs as compared to benign tissue. Differential expression of MED8 in renal cell carcinoma (RCC) and MED12 in lung cancer (LCa) were validated and further investigated by immunohistochemical staining on tissue microarrays containing large numbers of specimen. MED8 in clear cell RCC (ccRCC) associated with shorter survival and advanced TNM stage and showed higher expression in metastatic than primary tumors. *In vitro*, siRNA mediated MED8 knockdown significantly impaired proliferation and motility in ccRCC cell lines, hinting at a role for MED8 to serve as a novel therapeutic target in ccRCC. Taken together, our Mediator complex transcriptome proved to be a valid tool for identifying cancer-related shifts in Mediator complex composition, revealing that MEDs do exhibit cancer specific transcriptional expression profiles.

## INTRODUCTION

The Mediator, an evolutionarily conserved multi-protein complex, consists of 33 subunits (MEDs) in humans and is an indispensable regulator of the transcriptional machinery [1]. It can be divided into four distinct submodules: the head, middle, tail and kinase [2]. While the head, middle and tail modules form a stable core complex [3], the kinase module, consisting of the Mediator subunits MED12 and MED13, the cyclin-dependent kinase 8 (CDK8) and Cyclin C, associates reversibly with the core complex [4]. It strongly interacts with RNA polymerase II (Pol II), changes its conformation and influences the transcription initiation process as well as other important steps of protein expression [1–4]. Especially the kinase module is of high interest as it consists of subunits containing paralogs (MED12/MED12L, MED13/MED13L, CDK8/CDK19) which have arisen from gene duplications during vertebral evolution. The paralogs have been shown to form the kinase module leading to a high diversity in the quaternary structure of the Mediator complex, and may influence gene transcription essentially, yet the functions of these kinase subtypes are not well understood [5]. In human malignancies, several studies have already proposed an important role of the Mediator complex and its subunits in cancer development, progression and chemoresistance [6].

For example, altered MED1 protein expression has been reported for several cancer entities. Interestingly, MED1 is a transcriptional activator of several nuclear receptors, such as estrogen receptor (ER), thyroid receptor (TR), and androgen receptor (AR) [7]. In prostate cancer (PCa) MED1 overexpression was proposed to have implications in prostate oncogenesis through interaction with AR signaling [8]. Further, MED1 is partially overexpressed and plays a critical role in the development of tamoxifen resistance in breast cancer [9]. In contrast, downregulation of MED1 was described to promote the metastatic spread of human non-small-cell lung cancer [10] and triggers a tumorigenic phenotype in metastatic melanoma indicating context-specific MED profiles in different cancer entities [11]. Additionally, various genomic alterations were previously described. Especially *MED12* mutations frequently occur in uterine leiomyomas [12], phyllodes tumors [13] as well as in breast fibroepithelial tumors [14] and were also found in prostate adenocarcinoma [15]. The downregulation of *MED12* is associated with drug resistance in colon and lung cancer through regulation of TGF-β receptor signaling and induction of epithelial-mesenchymal transition, which emphasizes the various roles of MEDs and their interplay with other proteins in- and outside of the Mediator complex [16]. Only recently, overexpression of MED15 has been found in 76% of distant metastatic castration-resistant prostate cancer (CRPC) and in 70% of local-recurrent CRPC, in contrast to low frequencies in androgen-sensitive PCa and benign tissue [17].

Moreover, the inhibition of MED23 provokes a more aggressive phenotype in melanoma [18] and breast cancer [19]. In lung cancer patients, high MED23 was shown to be associated with worse outcome [20]. Especially in colorectal cancer, CDK8 is frequently described to serve as an oncogene that regulates β-catenin activity suggesting a potential therapeutic value for CDK8 in colorectal cancer patients [21]. Furthermore, this kinase subunit influences the progression of breast cancer [22, 23], gastric cancer [24] as well as melanoma [25].

Even though altered expression of several Mediator subunits has been shown in different malignancies, the transcriptional and protein expression profiles and possible clinical implication were reported for only a few subunits in certain cancer entities, e.g. MED1 in breast or CDK8 in colorectal cancer [9, 21]. In this study we therefore analyzed mRNA expression levels of the Mediator in 20 tumor entities from the Oncomine^™^ database yielding a comprehensive characterization of the tumor specific expression of MEDs in a cancer *vs*. benign analysis. Validation on protein level was performed by IHC for selected MEDs on tissue microarrays (TMAs). To our knowledge, this is the first systematic and comprehensive study deciphering the transcriptome of the whole Mediator complex across a large number of different cancer types.

## RESULTS

### Transcriptional expression of the Mediator

**Figure 1:**
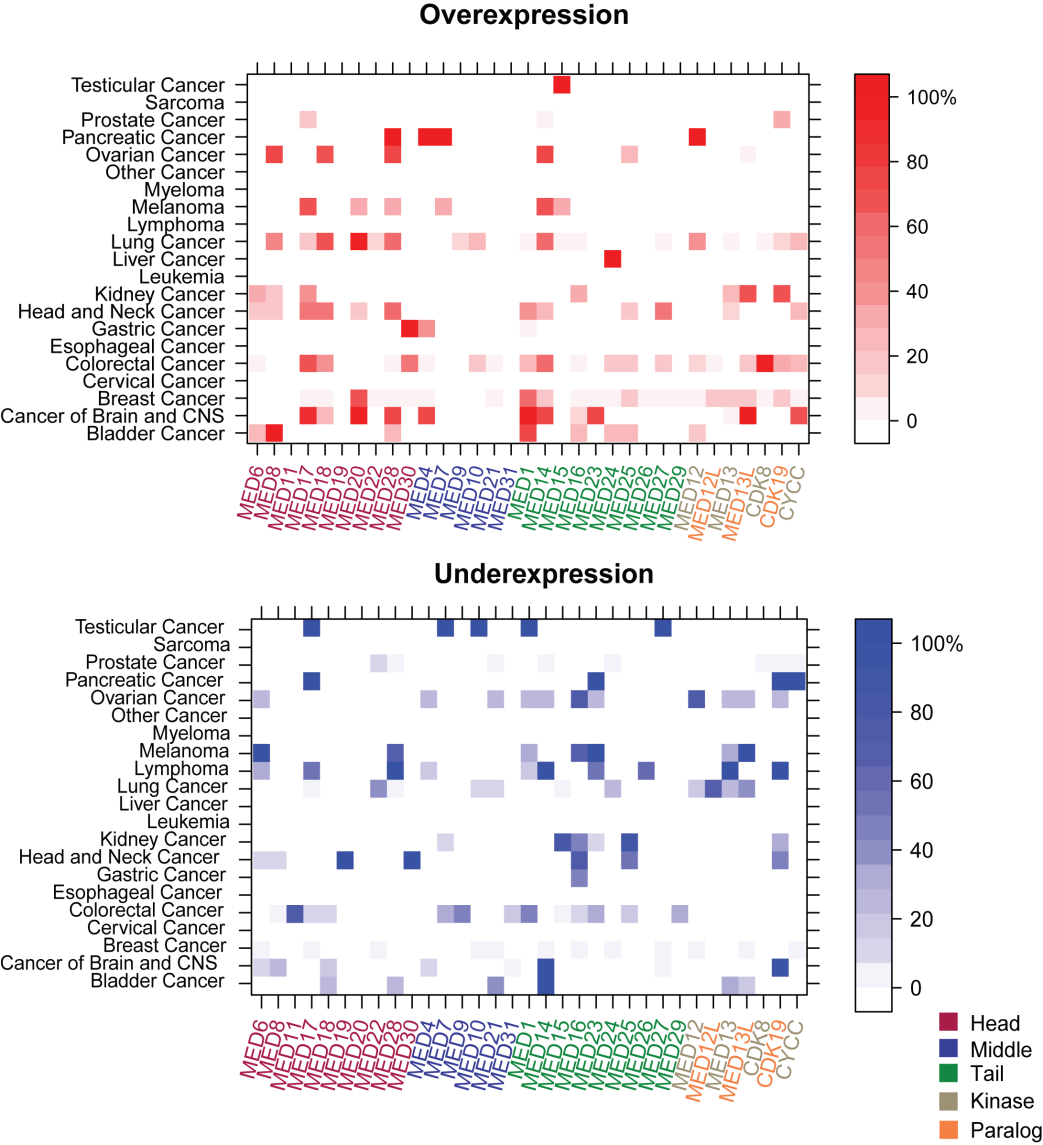
Transcriptional landscape of the Mediator complex in human cancers The levelplots depict the frequencies (%) of **A.** over- and **B.** underexpression for the Mediator complex subunits in all analyzed tumor entities. Mediator subunits (MEDs) are color-coded based on the modules which they are part of (head, middle, tail, and kinase with the paralogs). While in some cancer entities (cervical, esophageal cancer, sarcoma, myeloma and leukemia) no differential MED expression was observed, other tumors exhibited altered MED expressions as compared to benign reference tissue (e.g. lung and breast cancer). Red = overexpression. Blue = underexpression.

Using the Oncomine database we compared the mRNA expression in cancer *vs*. benign tissue. In total, 178,612 samples from 20 tumor entities were analyzed (Table S1). The results of the differential expression analyses are shown in Figure 1. Expression profiles differed considerably depending on the tumor entity analyzed.

#### Transcriptional expression regarding the tumor entities

While in some tumor entities only few MEDs were found to be differentially expressed in comparison with benign tissue, other entities differed distinctly in their MED expression patterns (e.g. lung cancer, head and neck cancer, colorectal cancer, cancer of brain and CNS). Lymphoma was found as the only tumor entity to solely show underexpression of its Mediator subunits (most strongly *MED28*, *MED14*, *MED13*, *CDK19*, all with a frequency of 100%, *n* = 11/11). In contrast, an isolated *MED24* overexpression was detected in liver cancer (100%, *n* = 99/99). In testicular cancer only a single overexpression (*MED15*) was found; while other subunits were underexpressed (*MED17*, *MED7*, *MED10*, *MED1*). While differentially expressed subunits in testicular (*n* = up to 23) and pancreatic tumors (*n* = 12) were found with a frequency of 100%; only low frequencies for over- (30% in *CDK19*, *n* = 121/404) and underexpression (8% in *MED22*, *n* = 23/290) were detected in prostate cancer. In colorectal cancer, the highest overexpression rate was found for *CDK8* with a frequency of 96% (*n* = 440/460). For the carcinoma of the breast, *MED1* and *MED20* exhibited the highest frequency rates (60%, *n* = 1688/2810, respectively 65%, *n* = 1778/2741). In kidney cancer, *MED8* showed an overexpression in 20% (*n* = 10/50) of all samples. In lung cancer, *MED12* was transcriptionally overexpressed in 36% (*n* = 226/628) and underexpressed in 21% (*n* = 132/628) of the samples. For the cancer entities sarcoma, myeloma, leukemia, esophageal, and cervical cancer, we did not observe differential expression of the Mediator subunits.

#### Transcriptional expression regarding the Mediator complex subunits

In a next step, we grouped the differentially expressed subunits of the Mediator complex by the modules which they are part of (head, middle, tail, kinase). As depicted in Figure 1A, the rate of overexpression is higher in the head, tail, and kinase as compared to the middle module, which showed almost no overexpression. In cancers of the head and neck neither over- nor underexpression of the middle module has been detected. In comparison, varied expression of the kinase module was found in several cancer entities (breast, colorectal cancer, and lung cancer).

In colorectal cancer, it is notable that the subunits of the kinase module, especially *CDK8*, exhibit enhanced expression (96%, *n* = 440/460). *MED12*, also a subunit of the kinase, was highly overexpressed in lung (36%, *n* = 226/628) and pancreatic cancer (100%, *n* = 10/10).

Upon closer examination of the individual subunits, for *MED8*, a subunit of the head module, high overexpression rates in ovarian (71%, *n* = 586/820), lung (47%, *n* = 226/483), bladder cancer (100%, *n* = 109/109) and renal cell carcinoma (20%, 10/50) were found. Furthermore, *MED1* was frequently overexpressed on mRNA level in breast cancer; an overexpression with a frequency of 60% (*n* = 1688/2810) was detected.

Certain MEDs were found to be both over- and underexpressed in the same cancer entity [e.g. *MED28* in bladder cancer (both 26%, *n* = 28/109); *CDK19* in kidney cancer (overexpression 66%, *n* = 33/50; underexpression 32%, *n* = 16/50)].

### Immunohistochemistry and functional investigations

To validate the transcriptional data obtained from the analysis of the Oncomine database, two tumors [renal cell carcinoma (RCC), lung cancer (LCa)] were selected for protein analysis (IHC) and functional analysis of either MED8 or MED12 respectively on large tissue microarray (TMA) cohorts with available clinical information and cell lines.

#### MED8 in RCC

**Figure 2:**
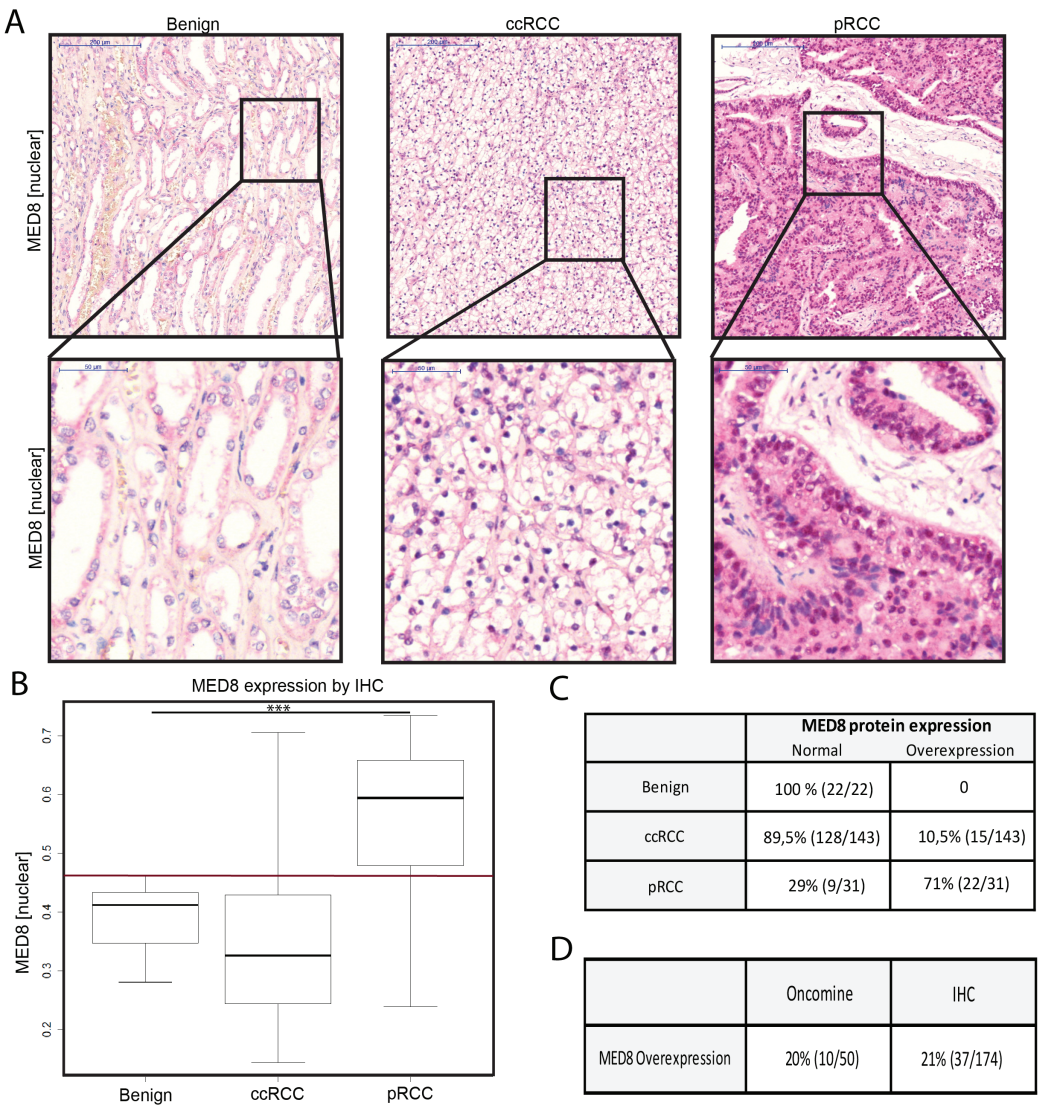
MED8 in RCC **A.** Representative IHC images from tissue of benign kidney, ccRCC and pRCC analysis of the MED8 protein expression with alkaline phosphatase as reporter dye (red), haematoxylin (blue) as counterstain. 5x (upper panel) and 40x (lower panel) objective magnification. **B.** MED8 protein expression profile of the total kidney cohort including benign tissue, ccRCC and pRCC. Boxplots of mean red chromogen intensity of the nucleus are shown. (Red reference line at y = 0.46 represents the cut-off for defining enhanced protein expression). **C.** Quantification of samples expressing MED8 in a normal and overexpressed range. **D.** Direct comparison between proportion of samples with a MED8 overexpression on mRNA (Oncomine) and protein level (IHC). (*** = *p* < 0.001, ccRCC = renal clear cell carcinoma, pRCC papillary renal cell carcinoma).

**Figure 3:**
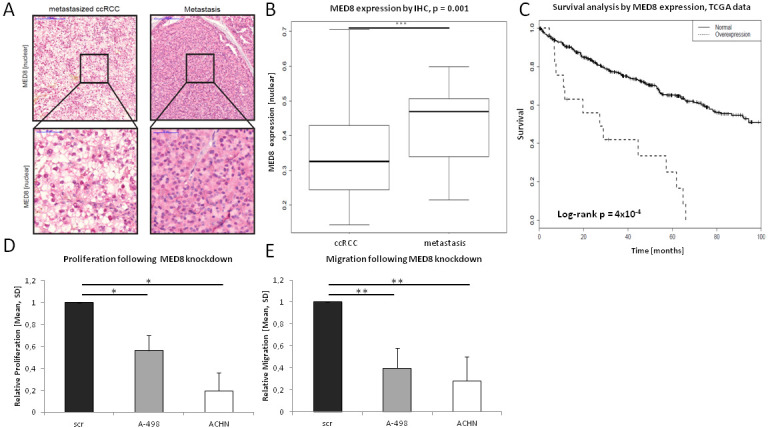
MED8 in ccRCC progression **A.**+**B.** Significantly enhanced MED8 protein expression was observed in metastases derived from ccRCC. **C.** MED8 overexpression (z-score > 1.5) is associated with strongly reduced survival in ccRCC patients. **D.** MED8 knockdown led to significantly reduced proliferation in the ccRCC cell lines A-498 and ACHN. **E.** Migration was significantly impaired in MED8-deficient cells as compared to scrambled control cells.

Protein expression of MED8 was found in both nuclear and cytoplasmic regions of the tissues analyzed (Figure 2A). In the RCC cohort, nuclear MED8 overexpression was detected in 21% (*n* = 37/174) (Figure 2B-2D). Most pronounced, the papillary RCC (pRCC) samples showed significantly higher nuclear MED8 protein expression as compared to benign renal tissue (*p* < 0.0001) and clear cell RCC (ccRCC) (*p* < 0.001). Interestingly, the expression of MED8 was significantly enhanced in metastatic ccRCC (Figure 3A+3B). When analysed independently for N- or M-stage, the differences remained non-significant, probably due to the low numbers of metastasized ccRCC (Figure S1A+B; Table 1). For further validation, the ccRCC TCGA cohort (*n* = 533) - an additional large mRNA expression cohort with available clinical data - was included into the analysis. In this, *MED8* was found to significantly associate with outcome related parameters of tumor severity such as lymphnode status, distant metastases and T stage (Figure S1D-F). Further, patients overexpressing *MED8* showed a strongly reduced survival (Figure 3C).

**Table 1:** Clinical pathological data of the kidney cohort

	RCC Σ=173	ccRCC Σ=142	pRCC Σ=31	Metastases Σ=30
**TNM**				
T1a	79 (45.7)	59 (41.5)	20 (64.5)	-
T2	38 (21.9)	32 (22.5)	6 (19.4)	-
T3	54 (31.2)	49 (34.5)	5 (16.1)	-
T4	2 (1.1)	2 (1.4%)	0 (0)	-
N1	9 (5.2)	8 (5.6%)	1 (3.2)	-
M1	21 (12.1)	18 (12.7)	3 (9.7)	-
Cancer associated death	13 (7.5)	11 (7.7)	2 (6.5)	
Relapse	34 (19.7)	31 (21.8)	3 (9.7)	
**Grading**				
G1	56 (32.4)	44 (31.0)	12 (38.7)	-
G2	111 (64.2)	94 (66.2)	17 (54.8)	-
G3	5 (2.8)	3 (2.1)	2 (6.5)	-

RCC: renal cell cancer, ccRCC: renal clear cell carcinoma, pRCC: papillary renal cell cancer

To further characterize the functional role of MED8 in metastatic spread and progression we performed *in vitro* assays with the ccRCC cell lines A-498 and ACHN. After siRNA mediated knockdown of MED8 (Figure S1H) proliferation and migration assays were undertaken. Proliferation was significantly decreased in the MED8-deficient ccRCC cell lines A-498 (*p* = 0.02) and ACHN (*p* = 0.01) (Figure 3D). Further, migration was significantly reduced in A-498 (*p* = 0.002) and ACHN (*p* = 0.002) cells following the transient MED8 knockdown as compared to control cells (Figure 3E). In conclusion, MED8 protein expression increased during the progression of ccRCC to metastatic sites and MED8 knockdown led to decreased malignant behavior in the ccRCC cell line A-498 and the metastatic ccRCC cell line ACHN.

#### MED12 in LCa

**Figure 4:**
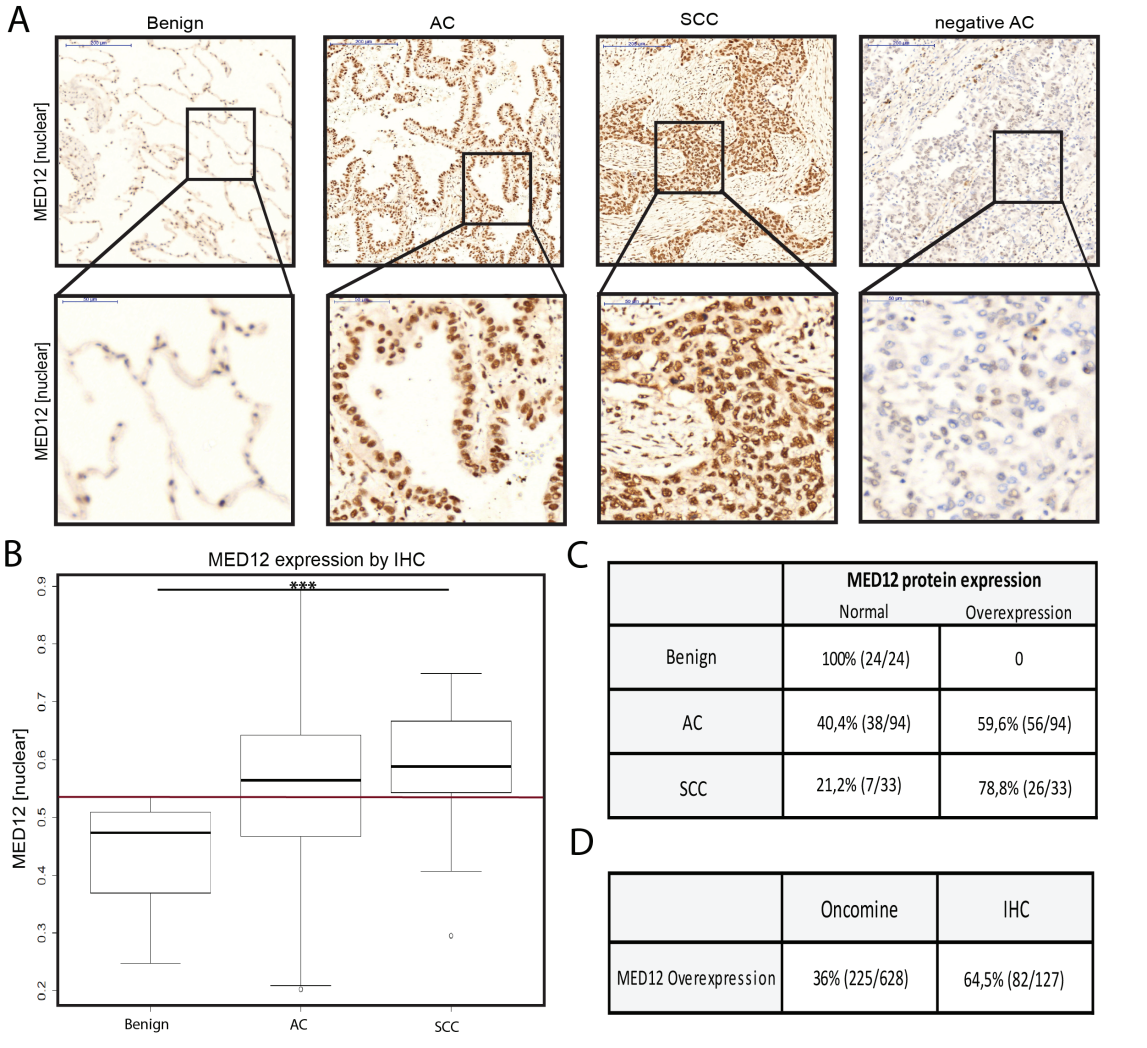
MED12 in LCa **A.** Representative IHC images from tissue of benign lung, AC and SCC analysis of the MED12 protein expression with haematoxylin. 5x (upper panel) and 40x (lower panel) objective magnification. **B.** MED12 protein expression profile of the total lung cohort including benign tissue, AC and SCC. Boxplots of mean brown chromogen intensity of the nucleus are shown. (Red reference line at y = 0.52 represents the cut-off for defining enhanced protein expression). **C.** Quantification of samples expressing MED12 in a normal and overexpressed range. **D.** Direct comparison between proportion of samples with a MED12 overexpression on mRNA (Oncomine) and protein level (IHC). (*** = *p* < 0.001, AC adenocarcinoma, SCC squamous cell carcinoma)

Protein expression of MED12 was found in nuclear and cytoplasmic regions of the tissues analyzed (Figure 4A). In total, 64.5% of LCa samples showed a nuclear overexpression of MED12 as compared to benign tissue (*n* = 82/127) (Figure 4B-4D). Both, lung adenocarcinoma (AC) and squamous cell carcinoma (SCC) exhibited a significantly elevated MED12 nuclear protein expression as compared to benign tissue (*p* < 0.001).

For MED12 in AC, no association of clinical-pathological parameters with MED12 protein expression was present, neither in the IHC cohort (Figure S2A-C) nor in the TCGA cohort (Figure S2D-F). Using the TCGA dataset for AC, the Kaplan-Meier estimator was used to split patients by under- *vs* normal expression of *MED12* in the long term. While underexpression was found to be associated with reduced survival, the log-rank value remained non-significant (Figure S2G).

*In vitro,* siRNA mediated knockdown of MED12 in the AC cell lines H1437 and H1792 (Figure S1I) did not influence proliferation as compared to scrambled control (Figure S2H). Interestingly, significantly enhanced migration was found in both AC cell lines, H1437 (*p* = 0.03) and H1792 (*p* = 0.007), following MED12 knockdown (Figure S2I).

## DISCUSSION

The Mediator complex serves as a hub for important signaling pathways [26] and it has became apparent, that deregulated MEDs link a multitude of different transcription factors, influence the tumorigenesis and tumor progression, and modulate drug sensitivity [6, 16]. Interestingly, deregulation and mutation of various MEDs are described to occur in different cancer types as factors, which either associate with tumor development or promote tumor aggressiveness such as metastatic spread [5, 11]. The kinase's main function is to regulate the whole Mediator and the transcription of a large number of protein-coding genes [27]. However, a detailed understanding on how the Mediator complex acts as a driver of carcinogenesis requires further studies for a more detailed understanding of the complexity of this multi-protein complex.

This study is the first to provide a comprehensive overview on the Mediator subunits expressed in a multitude of human cancers. By performing a transcriptome analysis of the Oncomine database, we identified distinct MEDs, which show tumor specific profiles and may influence the tumor behavior. Moreover, our analysis reveals that every single tumor entity has a specific transcriptional profile of the Mediator subunits.

Our analysis was able to confirm several recently published studies such as the overexpression of *MED1* in breast cancer at the protein level [9, 28] or the overexpression of subunits of the kinase module (especially CDK8) in colon cancer on the mRNA level [21]. Furthermore, a recently published study of our working group, which was deduced on the Oncomine transcriptome, was able to confirm the frequently occurring MED15 overexpression in testicular germ cell tumors on protein level (Figure 1) [29]. The IHC results provided additional evidence for the validity of the Oncomine-analysis based expression levels of the Mediator. It should be noted however, that there are limitations in comparing mRNA expression data with data from protein analyses (for reasons of technical limitations in sensitivity of low abundant mRNAs, as well as the different post-transcriptional modifications and regulations which influence mRNA stability/half life and thereby heavily influence protein levels in the cell).

Previously, diverse MEDs were described to be upregulated during tumor development and progression [6, 17]. In this study we provide additional evidence for cancer specific downregulation of certain MEDs, which may likewise be implicated in the disease and have great potential for further investigation. With the number of samples analyzed, our Oncomine database analysis represents a reliable source for future analysis. While our study mainly focused on the comprehensive study of the transcriptional regulation of the Mediator complex in cancer, ultimately it will be necessary to further characterize the Mediator complex at the protein level based on our results.

By taking a closer look at the transcriptome analysis it becomes apparent that several MEDs are found to be both over- and underexpressed dependent on the cancer entity (e.g. *MED28* in bladder cancer, *CDK19* in kidney cancer). This supports the hypothesis that heterogeneous molecular alterations contribute to cancer development and progression. A well investigated example for context-specific expression is MED1, which is upregulated in prostate and breast cancer [8, 9] whereas downregulation of MED1 was observed in lung cancer and aggressive melanoma [10, 11]. However, further investigations are needed to understand the role of the differentially regulated Mediator complex subunits on the molecular level.

For the validation of the mRNA results at the protein level, a selective combination of MEDs and cancer entities was made based on the results of the transcriptome analysis, the laboratory specific expertise for lung and urological tumors, availability of cohorts and commercially purchasable antibodies as well as previous published studies. For this selection only the results for overexpression were taken into account.

MED12 in LCa was chosen, because this Mediator complex subunit is frequently altered in diverse cancer entities [12–16] and was not yet investigated on a large lung cancer cohort with detailed clinical information. MED8 in RCC was selected, since only little is known about the MED8 subunit. Also, the role of the Mediator complex in RCC in general has not much been investigated yet [6]. In cancer, only the presence of *MED8* mutations in colorectal cancer cell lines has been described [28]. However, as we were not able to detect any over- or underexpression on the transcriptional level in colon cancer, we decided to validate MED8 expression on the protein level in RCC, for which no previous data was available. Interestingly, in RCC, we found a subentity-specific MED8 expression profile. Especially the pRCC samples showed significantly higher MED8 protein expression as compared to benign renal tissue and ccRCC (Figure 2). These subtype specific differences in expression levels are likely due to the different genetic backgrounds and the different behavior of the tumors - even though both arise from the proximal tubules.

Interestingly, MED8 in ccRCC was associated with the metastatic status in our kidney cohort, but remained non-significant probably due to low numbers of ccRCC samples with aggressive phenotype (Table 1, Figure S1A+B). In the next step, we therefore stained 30 metastases derived by ccRCC, which showed strongly elevated MED8 protein expression indicating a possible role for MED8 in metastatic spread (Figure 3A+3B). For increased statistical power, we decided to include TCGA datasets into our study to further investigate the clinical relevance of MED8 in ccRCC. Indeed, we were able to confirm that *MED8* is significantly associated with metastatic status, T stage and shorter survival time (Figure S1D-F, Figure 3C). It was these promising clinical associations for MED8 which led us to investigate the functional role of MED8 in ccRCC *in vitro*. Performing an siRNA mediated MED8 knockdown in the primary cell lines A-498 and the metastatic ACHN in ccRCC, we investigated its influence on proliferation and migration. Both ccRCC cell lines were significantly impaired in proliferation and motility following MED8 knockdown as compared to scrambled siRNA control cells, respectively (Figure 3D+3E). These findings are in accordance with the previously described data on MED8 expression, in which MED8 expression associated with worse disease outcome and TNM stage, respectively. In conclusion, MED8 might serve as a potential target in patients suffering from ccRCC to counteract tumor growth and metastatic spread.

Further, MED12 in LCa was found to be frequently overexpressed on the mRNA level, which was confirmed on the protein level (Figure 4A-4D). Underexpression (21% on mRNA level) was not further investigated due to the difficulties in defining the appropriate cut-offs for underexpression prior to performing immunohistochemistry - especially when expression of the benign reference tissue is already low. However, differential expression of MED12 in lung AC did not associate with TNM stage or survival which was available from the clinical data in the IHC cohort (Figure S2A-C). Nevertheless, as apparent from TCGA data, there was a tendency towards a worse outcome for lung AC patients underexpressing *MED12* in the long term, e.g. after 5 years (underexpression approximately 30% survivors *vs*. normal expression approximately 50% survivors) (Figure S2G). Low *MED12* expression showed a trend towards associating with positive metastatic status (Figure S2D) in patients suffering lung AC using the TCGA dataset. Further investigations for MED12 in AC *in vitro* revealed that proliferation did not differ between MED12 knockdown and scrambled control cells in both lung AC cell lines, namely H1437 and H1792 (Figure S2H). Interestingly, MED12 knockdown led to a strongly increased migratory potential in both cell lines suggesting a tumor suppressive role of MED12 in lung AC (Figure S2I). Previously, Huang et al. described, that cytoplasmic MED12 expression negatively regulates TGF-βR2 and that MED12 suppression leads to activation of TGF-β signaling [16]. Further, loss of MED12 was shown to induce a mesenchymal phenotype through activation of TGF-β signaling and thereby epithelial-mesenchymal transition (EMT), which is known to contribute in cancer progression and metastatic spread [16, 30]. Taken together, this data suggests that MED12 knockdown in the AC cell lines may lead to an EMT-like phenotype, which might explain the enhanced migratory potential (Figure S2A+I). Further functional investigations are needed to support this hypothesis.

In conclusion, we found MED8 to be frequently overexpressed in RCC patients. MED8 was associated with parameters of worse outcome and decreased survival in ccRCC. *In vitro*, inhibition of MED8 by transient knockdown led to decreased proliferation and motility in the ccRCC cell lines, potentially serving as a novel therapeutic target in patients suffering from ccRCC. Taken together, we have shown that unraveling the role of the Mediator complex in tumorigenesis and progression can help establishing novel tumor markers with medical value, e.g. for diagnostics, prognostics or therapy. The presented analysis of the Mediator transcriptome across human cancer entities may be a strong tool for selecting the relevant MEDs with greatest potential for further investigations.

## MATERIALS AND METHODS

### Ethics statement

Investigation has been conducted in accordance with the ethical standards and according to the Declaration of Helsinki and according to national and international guidelines. The study was approved by the local ethic committee (number: 121/13).

### RNA expression by oncomine

The database Oncomine^™^ (The Oncomine^™^ Research Edition) was utilized to investigate the mRNA expression profile of all 33 Mediator subunits. The Oncomine edition used allows free access to cancer *vs*. benign transcriptomic data of 20 tumor entities (Table S1). Oncomine^™^ (Compendia Bioscience^™^/LIFE Technologies) is a bioinformatics initiative aimed at collecting, standardizing, analyzing, and delivering transcriptomic cancer data for biomedical research [31]. Differential expression analyses (based on microarray studies) comparing the most prominent types of cancer with their respective benign tissues as well as a variety of cancer subtypes are available for exploration [32]. Datasets include metadata, which are used to set up analyses on groups of interest (cancer *vs*. benign, etc.). A simple global normalization strategy is applied to all datasets regardless of the platform or the pre-processing method. For mRNA data, this normalization consists of a log2 transformation as well as median centering. For our analysis of cancer *vs*. benign tissue we only considered primary tumors and the following cut-offs were applied in a pre-filtering step: p-value ≤ 0.05 (t-test) and fold change (FC) ≥ 1.5. This leads to the following definitions: overexpression = FC ≥ 1.5, underexpression = FC ≤ −1.5, 0 = FC < −1.5 to < 1.5. For the calculation of the frequency, the numbers of overexpressed respectively underexpressed samples were divided by the total number of analyzed samples.

### RNA expression by TCGA

RNA-sequencing data was imported from the Broad Institute Firehose Pipeline querying the standard data analysis run 2015-12-03 into R environment. Log2-transformed RSEM (RNA-Seq by Expectation Maximization) values per gene were used for expression analyses.

### Tissue microarray construction and protein expression analysis by immunohistochemistry (IHC)

Both, large kidney and lung TMA cohorts with comprehensive clinical information were used for protein expression analysis via IHC. The kidney cohort, provided by the Clinic for Urology of the University Hospital Bonn, contains 30 benign samples, 142 ccRCC samples, 31 pRCC samples, and 30 metastases derived from ccRCC (Table 1) [33]. The lung cohort, provided by the Institute of Pathology of the University Hospital Bonn, contains 30 benign samples, 100 AC samples and 35 SCC samples (Table 2).

**Table 2:** Clinical pathological data of the lung cohort

	LCa Σ=137 *n* (%)	AC Σ=102 *n* (%)	SCC Σ=35 *n* (%)
**TNM**			
T1	38 (27.7)	33 (32.4)	5 (14.2)
T2	85 (62.1)	61 (59.8)	24 (68.6)
T3	14 (10.2)	8 (7.8)	6 (17.2)
N +	44 (32.1)	32 (31.3)	12 (34.3)
**Grading**			
G1	5 (3.6)	5 (4.9)	0
G2	62 (45.2)	45 (44.1)	17 (48.5)
G3	70 (51.2)	52( 51.0)	18 (51.5)

LCa: lung cancer, AC: adenocarcinoma, SCC: squamous cell carcinoma

Prior to IHC of the kidney and lung cohort samples, tissue microarrays (TMAs) were constructed as described previously [34, 35]. Briefly, formalin-fixed paraffin-embedded (FFPE) tissues were cut into 4μm thick sections and mounted on slides. After staining with haematoxylin and eosin (H&E), relevant areas of benign tissue and primary tumor were identified and circled by a pathologist. Each tumor and corresponding benign region was represented with up to three cores measuring 0.6mm in diameter on a TMA recipient block using a semiautomatic tissue arrayer (Beecher Instruments, Sun Prairie, WI, USA). H&E TMA sections were assessed again to confirm the histology.

Prior to performing IHC analyses in the selected TMAs, the specificities of the antibodies were confirmed according to the manufacturer's instructions. Provided positive controls, which are placenta (MED8) and breast carcinoma (MED12), were tested and evaluated independently by two pathologists (SP, MB). IHC was performed using the Ventana Benchmark automated staining system (Ventana Medical System, Tuscon, AZ, USA). In brief, slides were incubated with the primary antibodies according to the manufacturer: anti-MED8 rabbit polyclonal (1:600, HPA028377, Sigma Aldrich, St. Louis, MO, USA) and anti-MED12 rabbit polyclonal (1:50, A300-774A, Bethyl Laboratories, Montgomery, TX, USA) at room temperature; antibody dilution was conducted using a Ventana diluent. For signal detection, the ultraView Universal DAB (lung) or ultraView Universal Alkaline Phosphatase Red (kidney) detection kit (Ventana Medical System, Tuscon, AZ, USA) was used. Finally, slides were counterstained with haematoxylin and bluing reagent, dehydrated, and mounted.

After performing IHC, the slides were digitized at 20x magnification using the Zeiss Panoramic Midi Scanner (3DHistech, Budapest, Hungary). IHC stainings were evaluated independently by two pathologists (SP, MB). Only cases with at least one assessable TMA core with sufficient tumor tissue were included in the analysis. Quantification of protein expression was performed using the semi-quantitative image analysis software Definiens (Tissue Studio v.2, Definiens AG, Munich, Germany), as described earlier [36]. Briefly, tumor or benign regions were selected manually for analysis. Each region was then analyzed with the software to measure its intensities [a continuous spectrum of average red staining intensity in arbitrary units (maximum range of readout 0.000-1.000)]. The Definiens software was used to analyze the average nuclear staining intensity (SI, corresponding to the mean brown/red chromogen intensity) quantified as a continuous value (arbitrary units) with higher values indicating stronger staining. The cut-offs used to determine a nuclear MED overexpression were consistently set above the expression of benign samples. The data were analyzed anonymously.

### Clinical data and statistics

Associations with clinical-pathological parameters were performed for ccRCC (kidney cohort) and AC (lung cohort). Survival analysis was evaluated by Kaplan-Meier estimator and log-rank tests. Statistical evaluation was performed using Student's t-test by Microsoft Excel, SPSS and R.

### Cell lines

All cell lines (Kidney: ACHN, A498; Lung: H1437, H1792,) were purchased from the American Type Culture Collection (ATCC^®^, Manassas, VA) and were grown in a 5% CO_2_ incubator at 37¼C and 85% humidity. Monolayer cultures were maintained in RPMI1640 (kidney cell lines) and DMEM (lung cell lines) medium (Biochrom) containing 10% heat-inactivated fetal calf serum (FCS, Sigma, St. Louis, MO), 1% streptomycin-penicillin antibiotics (Gibco^®^), and 1% glutamine (Thermo-Scientific Fisher, Darmstadt, Germany).

### SiRNA mediated *MED8* and *MED12* knockdown

Knockdowns were performed using pools of three distinct siRNAs each to target either MED8 or MED12 (MED8: sc-88195, MED12: sc-38595, Santa Cruz, TX, USA). A non-targeting scrambled siRNA was used as control (control siRNA: sc-37007, Santa Cruz, TX, USA). Transfections with 100 nmol/L siRNA were done using Screenfect A (Genaxxon Bioscience GmbH, Ulm, Germany). Efficient siRNA mediated knockdown of MED8 and MED12 was achieved 48 hours after transfection as confirmed by qRT-PCR (Figure S1H+I). Subsequently, knockdown experiments were started 48 hours post transfection.

### Quantitative reverse transcription PCR (qRT-PCR)

RNA was isolated from cell-line pellets using the Total RNA Purification Mini Spin Column Kit (Genaxxon Bioscience GmbH, Ulm, Germany). RNA quantity and quality was analyzed using a NanoDrop 2000 spectrophotometer (Thermo Scientific, Wilmington, DE, USA). cDNA was synthesized using 1 μg total RNA and the PrimeScript RT Reagent Kit with gDNA Eraser (Takara Bio, Saint-Germain-en-Laye, France). Quantitative real-time PCR was performed using 5 ng/μl cDNA, Takara Bio SYBR Premix Ex Taq II with ROX Plus and 10 pmol/μl forward/reverse primer. The following primer sequences were used: MED8 (forward 5′-GGCAGGTCAACCAGGGAAAA-3′, reverse 5′-TTCACTGCCCAACTCTGCAA-3′), MED12 (forward 5′-GGAGATTGAGGCTGAGCGTT-3′; reverse 5′-CAGCATGGGAGCCTGTGTAT-3′) and ß-Actin (forward 5′-CCAACCGCGAGAAGATGA-3′; reverse 5′-CCAGAGGCGTACAGGGATAG-3′). PCR was performed on an ABIPrism 7900 HT Fast Real-Time PCR System (Applied Biosystems, Foster City, CA, USA). Data were analyzed using Qbase+ (Biogazelle, Ghent, Belgium) with β-Actin (ACTB) as reference gene applying the 2−ΔΔCT algorithm. Statistical analysis was done in SPSS using the t-test (SPSS Statistics v21; IBM, Ehningen, Germany).

### EZ4U cell proliferation assay

The EZ4U cell proliferation assay kit was used following the manufacturer's recommendations (EZ4U; Biomedica Group, Vienna, Austria).

The siRNA transfections for proliferation assays were performed in 96-well plates. In each well of a flat-bottom 96-well plate, either 1.2×10^4^ cells (A498, H1792), or 2,4×10^4^ cells (ACHN, H1437) were seeded in 200 μl cell culture medium. MED8 and MED12 knockdown was then performed and cells were incubated to adhere and grow for 72 h. After incubation, 20 μl of EZ4U substrate solutions were added before incubating for about 3 h until the color of the solution changed from yellow to orange. The absorbance was measured using a micro-plate reader (Tecan, Model Spectra Thermo) at 450 nm wavelength.

### Migration assays

The siRNA transfections for migration assays were performed in 6-well plates. 48h post transfection, cells were trypsinized and seeded into migration boyden chambers. 5×10^4^ cells were plated in the upper chamber of migration inserts (VWR, Darmstadt, Germany) containing 0 % FCS medium. The lower chamber was filled with medium containing 10% FCS for chemotactic attraction. After 24 hours (lung cell lines) or 48 hours (kidney cell lines), cells were fixed with 4 % paraformaldehyde (Merck, Darmstadt, Germany), stained with haematoxylin (Waldeck, Münster, Germany) and washed with water. Membranes were scanned and manually evaluated by counting. Each experiment was repeated at least three times.

## SUPPLEMENTARY MATERIAL FIGURES AND TABLE




