# Supplementary material for: Comprehensive analysis of the transcriptional profile of the Mediator complex across human cancer types
Source: Oncotarget. 2016 Mar 30;7(17):23043–55. doi: 10.18632/oncotarget.8469 (PMC5029609; doi:10.18632/oncotarget.8469)
Supplement: Supplementary file 1 [file oncotarget-07-23043-s001.pdf]

Comprehensive analysis of the transcriptional profile of the mediator complex across human cancer types

Supplementary Material

Supplementary Table S1. Overview on the amount of over- [n(OE)] and underexpressed [n(UE)], as well as total [n(total)] number of samples analyzed. The table also lists the calculated frequency (%) for the Mediator complex subunits in the analyzed tumor entities which were obtained from the Oncomine database.

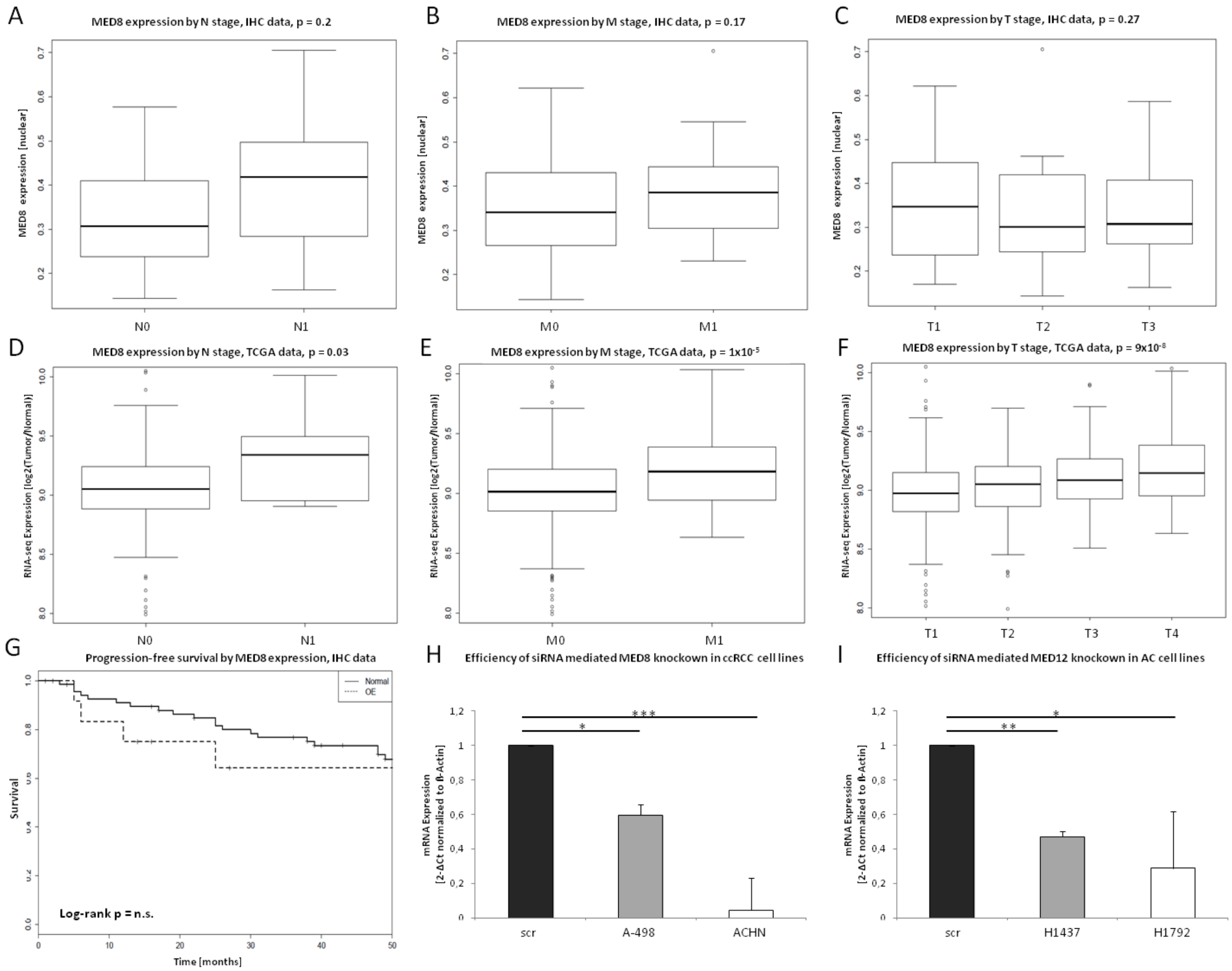

Supplementary Figure S1. Association of MED8 with parameters of worse disease outcome in ccRCC. A-C. MED8 protein expression did not significantly associate with N, M and T stage in the IHC kidney cohort. **D-F.** Based on the TCGA dataset, MED8 expression is found to be significantly associated with higher metastatic status (N+M stage) and T stage in ccRCC. **G.** Progression-free survival by MED8 overexpression in the kidney cohort. **H+I.** Efficiency of siRNA mediated MED8 knockown in ccRCC cell lines **(H)** and MED12 knockdown in AC cell lines **(I).**

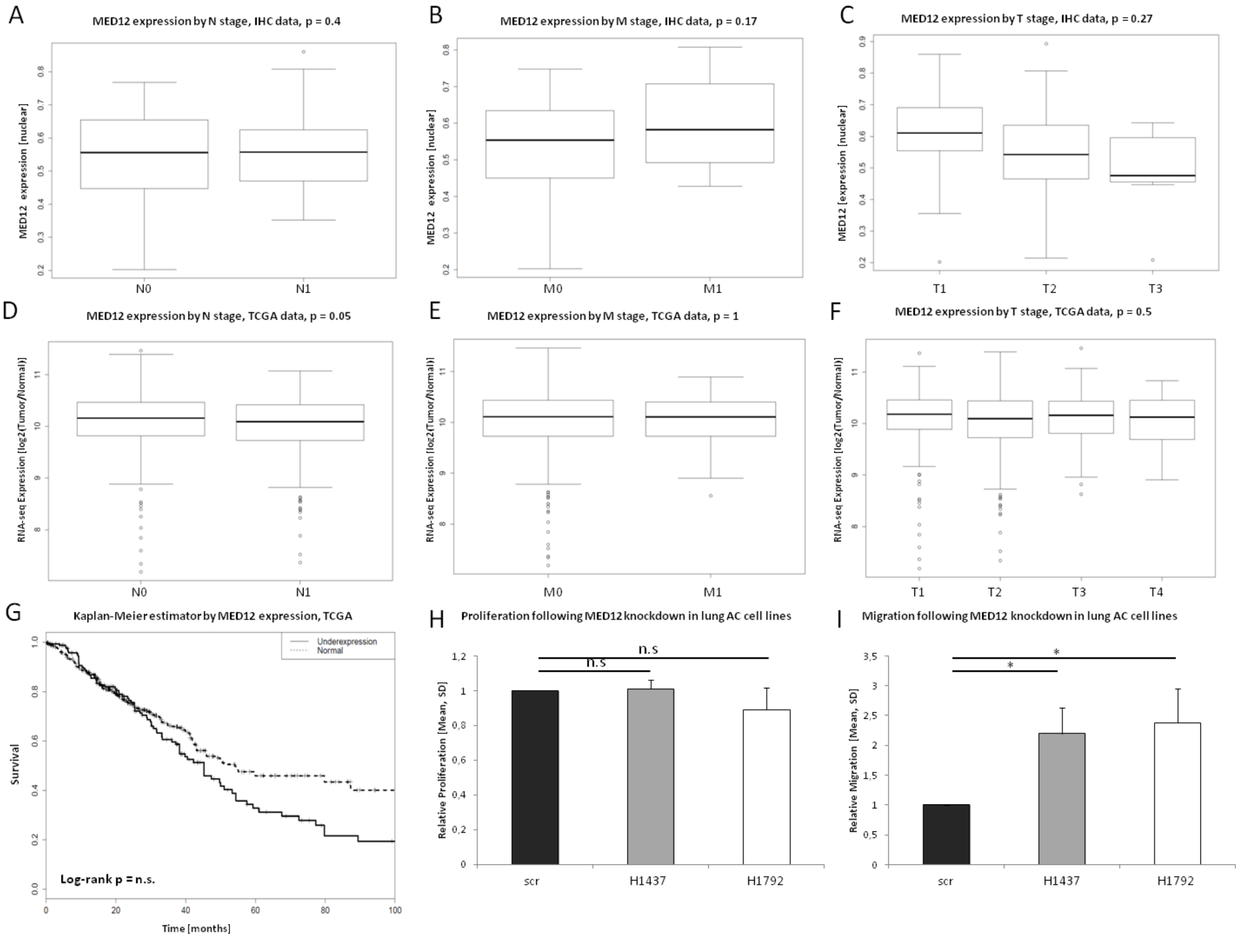

Supplementary Figure S2. Association of MED12 with parameters of worse outcome in AC. A-C. N, M and T stage were not associated with MED12 expression in the IHC lung cohort. **D-F.** MED12 underexpression is associated with positive lymphnode status in lung AC based on the TCGA dataset. No significant association was found in M- and T-stage. **G.** Survival analysis showed a tendency towards worse outcome for AC patients underexpressing MED12. Especially after 3-4 years, the Kaplan-Meier estimator was able to split the two groups using the TCGA dataset. **H+I.** Proliferation **(H)** of the lung AC cell lines H1437 and H1792 was not affected by siRNA mediated MED12 knockdown, whereas **(I)** Migration was strongly enhanced in MED12-deficient cells as compared to cells transfected with scrambled control siRNA.
